# Supramolecular Metal Halide Complexes for High-Temperature Nonlinear Optical Switches

**DOI:** 10.1021/jacs.3c13079

**Published:** 2024-02-23

**Authors:** Qian Wang, Jianbo Jin, Zhongxuan Wang, Shenqiang Ren, Qingyu Ye, Yixuan Dou, Sunhao Liu, Amanda Morris, Carla Slebodnick, Lina Quan

**Affiliations:** † Department of Chemistry, Virginia Tech, Blacksburg, Virginia 24061, United States; ‡ Department of Materials and Science Engineering, Virginia Tech, Blacksburg, Virginia 24061, United States; § Department of Chemistry, University of California, Berkeley, California 94720, United States; ∥ Department of Materials Science and Engineering, University of Maryland, College Park, Maryland 20742, United States

## Abstract

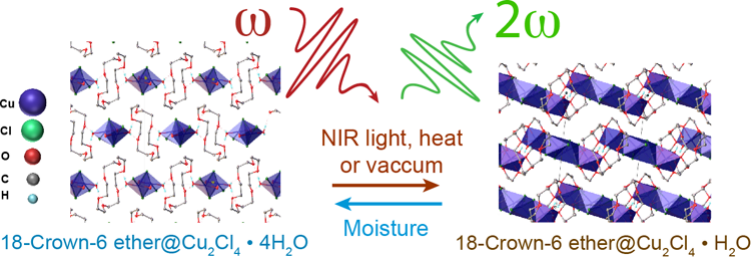

Nonlinear optical (NLO) switching materials, which exhibit reversible intensity modulation in response to thermal stimuli, have found extensive applications across diverse fields including sensing, photoelectronics, and photonic applications. While significant progress has been made in solid-state NLO switching materials, these materials typically showcase their highest NLO performance near room temperature. However, this performance drastically deteriorates upon heating, primarily due to the phase transition undergone by the materials from noncentrosymmetric to centrosymmetric phase. Here, we introduce a new class of NLO switching materials, solid-state supramolecular compounds 18-Crown-6 ether@Cu_2_Cl_4_·4H_2_O (**1·4H**_**2**_**O**), exhibiting reversible and stable NLO switching when subjected to near-infrared (NIR) photoexcitation and/or thermal stimuli. The reversible crystal structure in response to external stimuli is attributed to the presence of a weakly coordinated bridging water molecule facilitated by hydrogen bonding/chelation interactions between the metal halide and crown-ether supramolecules. We observed an exceptionally high second-harmonic generation (SHG) signal under continuous photoexcitation, even at temperatures exceeding 110 °C. In addition, the bridging water molecules within the complex can be released and recaptured in a fully reversible manner, all without requiring excessive energy input. This feature allows for precise control of SHG signal activation and deactivation through structural transformations, resulting in a high-contrast off/on ratio, reaching values in the million-fold range.

## Introduction

Nonlinear optical (NLO) switching phenomena refer to changes in the NLO properties of materials in response to external stimuli such as electromagnetic field, heat, pH variations, and pressure.^1−8^ In conventional linear optics, the response of a material to light is directly proportional to the intensity of the incident light. However, in NLO optics, the response goes beyond linear relationships and involves higher-order effects, such as second-harmonic generation (SHG).^9,10^ In solid-state NLO switches, the switchable nonlinear response is achieved by breaking the chemical bonds, tuning the molecular configurations, or harnessing the misalignment of dipoles. The switching mechanism is achieved in hybrid metal halides,^6^ metal–organic frameworks,^11^ host–guest inclusion systems,^12^ ferroelectric materials,^13−16^ and polymer compounds^17^ through structural phase transitions triggered by thermal stimuli, resulting in a transformation from a noncentrosymmetric structure (SHG-active state) to a centrosymmetric structure (SHG-silent state). A common trend among these materials is the switching-off of SHG intensity upon heating, owing to the restoration of the inversion center from a noncentrosymmetric structure.^16,18,19^ Furthermore, most of these solid-state crystalline materials exhibit low-contrast optical nonlinearities, typically below 100; examples include K_*x*_(NH_4_)_2–*x*_PO_3_F,^20^ (Me_3_NNH_2_)_2_[CdI_4_],^8^ and [C_4_H_10_N][CdCl_3_].^12^ This observation prompted us to investigate high-contrast NLO switching materials capable of functioning within a high-temperature range suitable for applications under extreme conditions.

Supramolecular host compounds are emerging as strong candidates for assembling host–guest compounds.^21−24^ This is attributed to their adaptable cavities and captivating coordination capabilities with various metal cations,^25−29^ including transition metal and alkali metals. These unique features are attributed to their multiple donor sites provided by heteroatoms such as oxygen and nitrogen.^23,24^ As a result, diverse intricate building units with remarkable optical properties can be formed.^30−34^ Notably, 18-crown-6 ether serves as a host by using its six oxygen atoms with lone-pair electrons to anchor suitable guest molecules through cationic or hydrogen bonding interactions.^16,35−38^ This approach capitalizes on the controllable molecular dynamics of polar states in host–guest systems, allowing for the tuning of their structural transformations.

We synthesized two solid-state single-crystalline crown-ether-metal-halide complexes, **18-Crown-6 ether@Cu**_**2**_**Cl**_**4**_**·4H**_**2**_**O** (denoted as **1·4H**_**2**_**O**) and **18-Crown-6 ether@Cu**_**2**_**Cl**_**4**_**·H**_**2**_**O** (denoted as **2·H**_**2**_**O**). In both complexes, copper chloride building blocks coordinate with the crown ether through either hydrogen bonding interactions (**1·4H**_**2**_**O**) or a combination of hydrogen bonding and direct chelation of the crown ether to copper(II) (**2·H**_**2**_**O**). Upon subjecting **1·4H**_**2**_**O** and **2·H**_**2**_**O** to NIR light excitation or thermal energy, we observed an efficient NLO effect characterized by SHG. The SHG intensity strongly depends on NIR light illumination time duration and power density, which can be attributed to the structural transformation from a centrosymmetric to a noncentrosymmetric structure through the release of water molecules during the photoinduced thermalization process. In addition, the water molecules in **1·4H**_**2**_**O** can be reversibly released and captured by controlling the humidity, enabling precise control over the quenching and activation of the SHG signal with an extremely high-contrast off/on ratio on a scale of millions. Moreover, **1·4H**_**2**_**O** exhibits strong broadband white downconversion photoluminescence (PL) emission and a long lifetime at room temperature (RT), attributed to self-trapped excitons (STE). Our work presents a novel approach for manipulating the NLO effect through the control of bridge water molecules in supramolecular metal halide complexes, opening new possibilities for NLO switching and potentially high-temperature, responsive ferroelectric applications.

## Results and Discussion

### Structural Analysis of Supramolecular Metal Halide Complexes

Here, we synthesized two single crystals using a facile solution processable approach. The first compound, **1·4H**_**2**_**O**, with a blue single crystal, was grown using a slow evaporation method at RT. The second compound, **2·H**_**2**_**O**, was synthesized using a similar approach but in an inert atmosphere with extremely low moisture content, and it exhibits a brown color. Single-crystal X-ray diffraction (SCXRD) characterization revealed that both **1·4H**_**2**_**O** and **2·H**_**2**_**O** crystallize in the monoclinic, centrosymmetric space group *P*2_1_/*n* at 100 K. Crystallographic details can be found in Tables S1 and S6. The uniform phase purity of the materials was confirmed by powder X-ray diffraction (PXRD) on the bulk crystals (Figure S1).

#### Structure Description of **1·4H**_**2**_**O**

**Figure 1:**
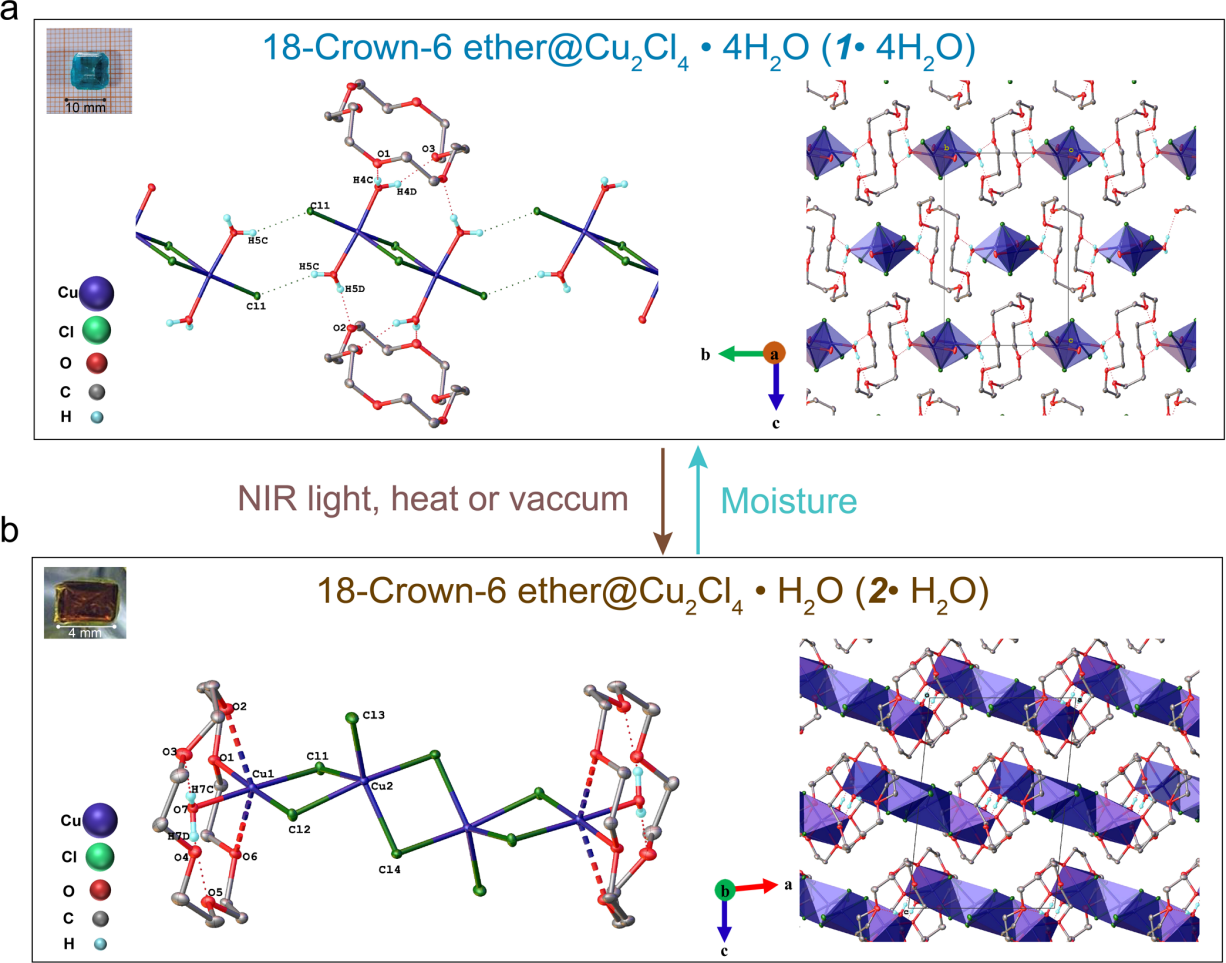
Single-crystal structures of compounds **1·4H_2_O** and **2·H_2_O** along with illustrations depicting their reversible structural transformations upon external stimuli. (a) Left: **1·4H**_**2**_**O** depicting the Cu_2_Cl_4_(H_2_O)_4_ dimer and H-bonding interactions with the crown ether (crown ether H atoms are omitted for clarity). Right: Packing diagram viewed down the *a*-axis showing the projection of the H-bonding layers into the page and running parallel to the *b*-axis. (b) Left: **2·H**_**2**_**O** molecule depicting the H-bonding interactions (crown ether H atoms omitted for clarity). Right: Packing diagram viewed down the *b*-axis.

Figure 1a illustrates the single-crystal structure of **1·4H**_**2**_**O**. The inorganic component comprises dimeric Cu_2_Cl_4_(H_2_O)_2_ complexes that sit on an inversion center. The Cu ions are in the +2 oxidation state and adopt a distorted trigonal bipyramidal geometry with three Cl atoms in the equatorial plane, two of which bridge the Cu atoms. The axial positions are occupied by the two water molecules. The water molecules of the dimer are hydrogen bonded with neighboring dimers (O5–H5C···Cl1) and 18-crown ether-6 (O4–H4C···O1; O4–H4D···O3; and O5–H5D···O2) to form 2D H-bonding layers running parallel to the (001) crystallographic plane. The packing diagram of Figure 1a is viewed down the *a*-axis with the H-bonding plane projected into the plane of the page and running parallel to the *b*-axis. There is an alternate dimer-crown ether pattern along the *b*-axis. The layers are stacked along the *c*-axis such that the Cu dimer and crown ether positions are staggered.

#### Structure Description of **2·H**_**2**_**O**

This structure has been discovered previously through a different synthetic method.^39^ Compound **2·H**_**2**_**O** forms isolated molecules comprising tetrameric (CuCl_2_)_4_**·**2H_2_O sandwiched between two crown ethers (Figure 1b). The Cu ions are in the +2 oxidation state, and the tetramer has inversion symmetry with two crystallographically unique Cu atoms. The copper ions in the core (Cu2) are five-coordinate, each with four bridging and one terminal chloride. The peripheral copper ions (Cu1) are six-coordinate Jahn–Teller distorted octahedra. The shorter bonds in the equatorial plane are filled by two bridging chlorides, O7 from water and O1 from the crown ether. Atoms O2 and O6 of the crown ether form longer axial bonds to Cu1. The atoms of the crown ether that are not directly bonded with Cu form hydrogen bonds with the O7 water molecule (O7–H7C···O3 and O7–H7D···O4). The packing diagram of **2·H**_**2**_**O** is viewed down the *b*-axis. The closest intermolecular contacts are nonbonding interactions between the chloride atoms and −CH_2_ groups of the crown ether with the shortest nonbonding distance being Cl4···H7A-C7 at 2.6189(4) Å.

#### Thermal-Induced Structural Transformation

**Figure 2:**
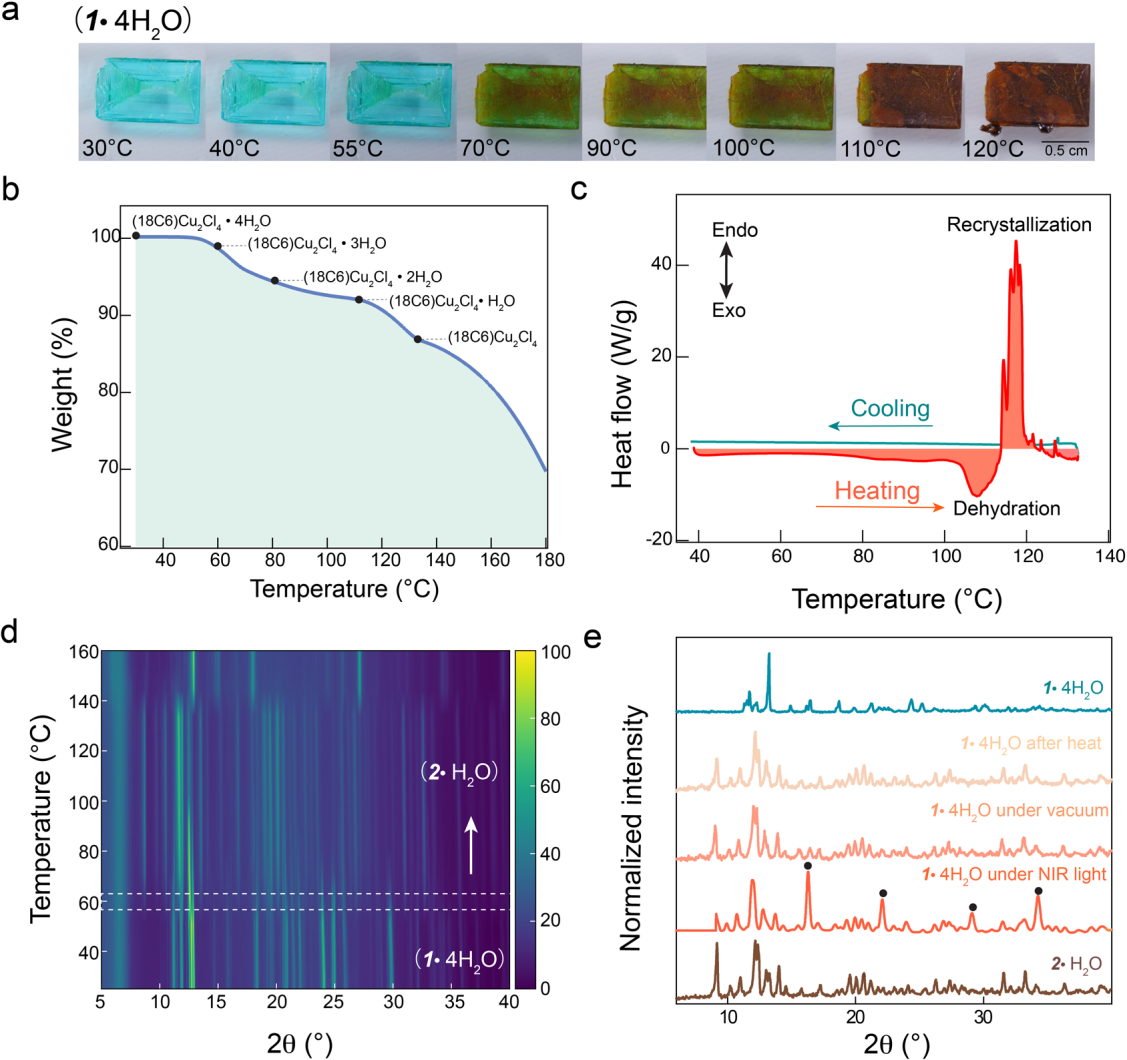
Structural analysis under varied external stimuli. (a) Images of the **1·4H**_**2**_**O** single crystal under elevated temperatures. (b) TGA curve of the **1·4H**_**2**_**O** single crystal. (c) DSC curve for **1·4H**_**2**_**O.** The orange and blue lines indicate the heating and cooling processes, respectively. (d) In situ temperature-dependent PXRD measurement on ground powder of **1·4H**_**2**_**O**. (e) Stimuli-dependent PXRD measurements on **1·4H**_**2**_**O** single crystals and a comparison with **2·H**_**2**_**O**.

The thermal-induced structural transformation of **1·4H**_**2**_**O** was studied via thermal gravimetric analysis (TGA), differential scanning calorimetry (DSC), PXRD measurements, and SCXRD analysis. We have observed a striking color change in the crystal, shifting from light blue to dark brown as the temperature increased from RT to 120 °C (Figure 2a). We initially performed TGA measurements to investigate the dynamic process of structural transformation as a function of temperature, as indicated in Figure 2b. By employing the weight loss percentage calculation, we estimated the compositions, with a primary emphasis on the changes in the number of water molecules. We observed a gradual loss of four water molecules occurring between approximately 50 and 120 °C. Notably, there are no distinct inflection points in the TGA curve within the temperature range of approximately from 50 to 110 °C, indicating an absence of a clear sequential and preferential loss of the first three water molecules from **1·4H**_**2**_**O** and gradual transition to a new structure. However, a slight inflection point appears near 118 °C, corresponding to the onset of loss of the final water molecule in monohydrated **1·4H**_**2**_**O**, ultimately resulting in the formation of the dehydrated structure. This interpretation of the TGA data aligns with the TGA results for **2·H**_**2**_**O**, which shows the loss of a single water molecule between ∼118 and ∼130 °C (Figure S2a). At 130 °C, the crown ether begins to decompose.

The DSC result of **1·4H**_**2**_**O** in Figure 2c shows a consistent heat capacity during the heating process up to ∼118 °C, at which point there is a significant decrease in heat capacity, indicating the recrystallization process from monohydrate **1·4H**_**2**_**O** to the new structure, i.e., brown **2·H**_**2**_**O**, accompanied by the loss of water molecules. This transformation is further supported by the absence of distinct peaks in the DSC curve of **2·H**_**2**_**O** during the heating process. However, a slight increase in heat capacity is noticed after 118 °C, attributed to the disruption of hydrogen bonds during the loss of water molecules within the crown ether in **2·H**_**2**_**O**, as illustrated in Figure S2b.

Through SCXRD analysis, we observed that the heated sample shares the same crystal structure as **2·H**_**2**_**O** (see Table S5 for crystal data on 120 °C annealed **1·4H**_**2**_**O**). SCXRD studies conducted at −173, −50, 25, and 55 °C indicate that the primary species is **1·4H**_**2**_**O,** exhibiting a thermal volume expansion of 3.4% (Tables S1–S4). Complementary temperature-dependent PXRD studies in Figure 2d were performed to assess the compound’s structural evolution across temperatures ranging from RT to 160 °C. Until approximately 55 °C, only the presence of **1·4H**_**2**_**O** was evident. In the temperature range of ∼55 to ∼110 °C, PXRD analysis revealed a mixture of thermally stimulated derivatives resulting from the stepwise loss of water molecules from **1·4H**_**2**_**O**. Between ∼110 and ∼140 °C, the structure underwent a transformation, becoming identical to that of **2·H**_**2**_**O**.

Based on the in situ temperature-dependent PXRD and TGA-DSC properties of **1·4H**_**2**_**O** between 55 and 130 °C, we propose the following mechanism of transition from a single-crystalline state of **1·4H**_**2**_**O** to a powdery state, subsequently undergoing recrystallization into **2·H**_**2**_**O** and further to dehydrated crystals. Dehydration initiates at approximately 55 °C with the loss of the O5 water molecule, which has the weakest O5–H5C···Cl1 hydrogen bond in the structure (as depicted in Figure 1a). Upon the loss of this water, Cl1 from an adjacent dimer migrates to occupy the vacant coordination site. This structural adjustment has multiple consequences, including the loss of symmetry (including the inversion center), partial amorphization, and strain/weakening of the remaining hydrogen bonds. The weakening of these remaining hydrogen bonds triggers an allosteric effect, facilitating the loss of more water molecules. The removal of the O5 water molecule from the neighboring dimer and the movement of Cl1 into the open site leads to the formation of the two central atoms in **2·H**_**2**_**O**, which involves bridging Cl atoms (corresponding to Cu2 and Cl4 in Figure 1b). The loss of the two O4 water molecules bound to these central coppers in **1·4H**_**2**_**O** completes the formation of the five-coordinate copper centers. Concurrently, the terminal Cu atoms each release the O5 water molecule, and the crown ether swings in, binding to the exposed site on the Cu. The remaining water and Cu correspond to the O7 and Cu1 in Figure 1b. Only after this entire transition is completed is inversion symmetry and crystallinity reestablished. The amorphization observed during the water loss and rearrangement process explains why the only two crystalline phases identified during the heating process between 25 and 118 °C are **1·4H**_**2**_**O** and **2·H**_**2**_**O**. Furthermore, both phases are present over a significant temperature range in PXRD (as shown in Figure 2d). When the heating is slow enough and sufficient annealing time is provided, this process proceeds as a single-crystal-to-single-crystal transition. It is worth noting that with the loss of the final water molecule from **2·H**_**2**_**O** to the dehydrated crystals between 118 and 130 °C, the loss of inversion symmetry and amorphization are likely to occur once again.

#### Structural Transformation under Vacuum, Heat, and NIR Photoexcitation

We then conducted a study to investigate the structural transformation of **1·4H**_**2**_**O** under diverse external stimuli, including heat, vacuum, and NIR photoexcitation. Interestingly, we observed that subjecting **1·4H**_**2**_**O** to high vacuum, 120 °C annealing, or exposure to NIR laser illumination led to a transformation in which the crystals changed color to brown. These intriguing findings prompted us to explore whether applying these stimuli to **1·4H**_**2**_**O** resulted in dehydration and the formation of **2·H**_**2**_**O**. To test this hypothesis, we performed PXRD analysis (Figure 2e), which confirmed that the dehydration and structural transition of **1·4H**_**2**_**O** to **2·H**_**2**_**O** occurred under all of these stimuli. For the sample illuminated with the NIR laser, the inhomogeneous distribution of power density on the sample area resulted in the formation of the degradation product CuCl_2_·2H_2_O (Figure S3). However, this does not impact the NLO switching property of the material, as CuCl_2_·2H_2_O exhibits negligible in NLO properties (Figure S4).

### Nonlinear Optical Properties

Given the transformation of the structure of **1·4H**_**2**_**O** from a centrosymmetric to a noncentrosymmetric configuration under external stimuli, we proceeded to examine the material’s NLO properties under in situ NIR laser illumination. The NLO response can be influenced by various factors, including the interactions of the incident photons with the material’s electronic structure as well as its inherent nonlinear susceptibility. High power pulsed laser excitation in noncentrosymmetric crystals results in higher-order phenomena, such as SHG, which produces photons with twice the energy of the incident ones. Initially, we employed a relatively low laser power density (excitation λ = 1060 nm at a pump power density of 764 μJ/cm^2^) to measure the SHG of **1·4H**_**2**_**O**. Our attention was piqued when we observed an increase in the SHG signal as a function of NIR illumination time, ultimately leveling off at an exceptionally high SHG switching contrast of approximately 3 × 10^7^ (Figure S4). We propose that this illumination process causes thermal dehydration, which leads to a transformation from a centrosymmetric to a noncentrosymmetric configuration, as described earlier in the proposed mechanism for the transformation from **1·4H**_**2**_**O** to **2·H**_**2**_**O** and further to anhydrous powders. These changes are also evident in the polarization resolved anisotropy SHG results (Figure S5), which can be attributed to the surface effect with two types of rotational symmetry, namely, 4-fold and 2-fold symmetry.^40−42^

**Figure 3:**
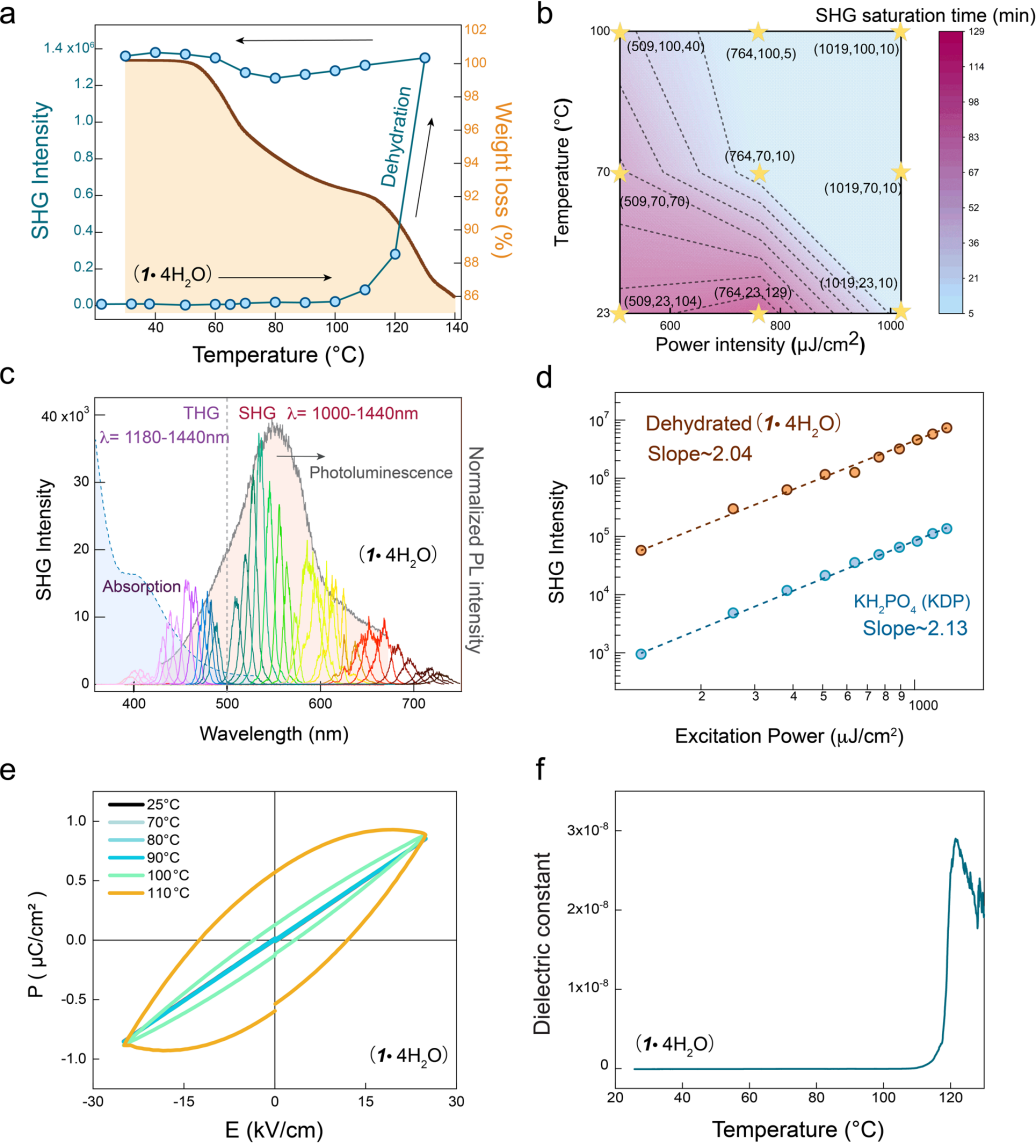
Nonlinear optical and electrical characterization of **1·4H**_**2**_**O.** (a) Temperature-dependent SHG evolution (blue) under an excitation wavelength of 1060 nm and corresponding TGA curve (brown). (b) Two-dimensional contour plot of the in situ time-dependent SHG intensity of **1·4H**_**2**_**O** and corresponding changes by altering excitation laser power density and temperature. The axis of yellow stars represents the numerical value of excitation power densities (μJ/cm^2^), temperature (°C), and SHG saturation time (min), respectively. (c) Combined spectrum of absorption, photoluminescence, and excitation wavelength-dependent SHG and THG spectrum from **1·4H**_**2**_**O**. The peak positions below 500 nm represent the THG signals, and the peaks above 500 nm are the SHG signals. (d) Excitation power-dependent SHG and fitted quadratic power law for **1·4H**_**2**_**O** and KDP at λ_pump_= 1060 nm. (e) Temperature-dependent P-E hysteresis loop shows the polarity in **1·4H**_**2**_**O**. (f) Temperature-dependent dielectric constant at 10 kHz.

We subsequently conducted an in situ temperature-dependent SHG measurement to understand the SHG enhancement under NIR illumination on a home-built optical setup (Figure 3a). Based on the crystal structure analysis, we attribute the loss of inversion symmetry to a slight increase in the SHG signal observed between 50 and 100 °C during heating. With progressing thermal-induced water loss, the sample gains more noncentrosymmetric character, which corresponds to a gradual augmentation in the SHG signal, as illustrated in Figure S6. Starting at around 110 °C, the structurally transformed heterophase and surface effects lead to a sudden elevation of SHG intensity to 1.4 × 10^6^ at 509 μJ/cm^2^, eventually resulting in the sample’s transition to anhydrous form at 130 °C. Upon cooling, this signal remains relatively stable, showcasing structural resilience. Additionally, we conducted electrical polarization measurements to provide further evidence of the polarization change in **1·4H**_**2**_**O** at elevated temperatures (Figure 3e,f and Figure S7). These measurements encompassed both the polarization-electric field (P-E) hysteresis loop and the temperature-dependent dielectric curve, highlighting the emergence of polarizability as the temperature increased from RT to 110 °C.

Moreover, since the sample of **1·4H**_**2**_**O** annealed at 120 °C gives the same structure as the brown **2·H**_**2**_**O** crystal, we also investigated the NLO behavior of **2·H**_**2**_**O** under long-duration NIR excitation. Surprisingly, we also observed the time-dependent SHG enhancement and quadratic slope of SHG with a high laser-induced damage threshold (LIDT) value of 2165.6 μJ/cm^2^ in **2·H**_**2**_**O**, as shown in Figure S8. Thus, even with the presence of a single water molecule in the crystallographic asymmetric unit, **2·H**_**2**_**O** can still undergo dehydration induced by photoinduced thermal energy under continuous NIR laser illumination, indicating that the potential saturated SHG signal plateau is derived from the fully dehydrated **1·4H**_**2**_**O** before crown-ether degradation.

We next investigated the synergistic effects of excitation pump power density and temperature, as shown in Figure 3b and Figure S9. The SHG signal was measured at three excitation power densities (509, 764, and 1019 μJ/cm^2^) and three temperatures (22, 70, and 100 °C). We observed a shorter saturation time and enhanced SHG intensity at higher temperature and pump power intensity. For instance, at RT, it took approximately 104 min to stabilize the SHG intensity when the power density was set at 509 μJ/cm^2^, while this saturation process was shortened to approximately 70 and 40 min at 70 and 100 °C, respectively. When the temperature was held at a constant of 22 °C and the pump power density was increased to 764 μJ/cm^2^, additional SHG growth was observed with a time dependence before reaching the plateau. Further elevating the power to 1019 μJ/cm^2^ at 22 °C led to the additional amplification of the SHG signal without significant time dependence. This implies that increasing the pump power to 1019 μJ/cm^2^ can directly transition the structure to its SHG saturation state without undergoing a time-dependent slope at 22 °C. When maintaining a constant temperature of 70 or 100 °C and incrementally increasing the pump power from 509 to 764 μJ/cm^2^ and subsequently to 1019 μJ/cm^2^, the SHG signal escalated on each occasion and reached a plateau without time dependence. These findings underscore the combined effect of temperature and laser power density in achieving a stable phase of **1·4H**_**2**_**O** with a high SHG contrast and shortening the saturation time.

We then examined the excitation wavelength dependence of the NLO measurements. By varying the excitation laser wavelength from 1100 to 1440 nm with a step of 20 nm shown in Figure 3c, we observed the entire NLO responses of the laser-saturated **1·4H**_**2**_**O** derivative across the whole visible absorption and emission spectra. Each incident laser light with frequency ω was upconverted into two distinct signals with frequencies of 3ω and 2ω, corresponding to the THG and SHG, respectively. Additionally, the SHG and THG signals exhibit quadratic and cubic dependences, respectively, on the pump power, as shown in Figure 3d (and Figure S10 for THG). Importantly, the laser-activated **1·4H**_**2**_**O** exhibits a strong SHG signal with a high LIDT under an excitation power of 2127.3 μJ/cm^2^, and the integrated intensity reaches approximately 4.8 × 10^7^ (Figure S11). This finding further indicates that the **2·H**_**2**_**O** crystal behaves similarly to **1·4H**_**2**_**O** after continuous laser excitation. In summary, our comprehensive investigations confirmed that the intense visible signals emitted by compound **1·4H**_**2**_**O** after prolonged laser illumination were indeed the result of high harmonic generation.

We also conducted diffused reflectance (Figure S12) and photoluminescence (PL) spectroscopy of **1·4H**_**2**_**O** (Figure S13). The THG signal corresponds closely to the absorption wavelength range, reinforcing the efficiency of the third harmonic generation in the nonlinear optical medium. However, a notable distinction was identified at the band-edge wavelength, where the THG signal intensity was enhanced, while it was attenuated at the absorption peak. For the SHG signal, we found a significant congruence between the spectral envelope of the SHG and the broad STE spectrum from the PL measurements. This alignment signifies efficient SHG within the crystal, facilitated by phase matching where two photons at the fundamental wavelength are synchronized to merge into one photon at the second harmonic wavelength. These signal alignments and improvements at the absorption band edge and emission peak position can be elucidated by the resonance enhancement effect,^43−45^ which comes into play when the energy of the second (or third) harmonic coincides with an electronic transition in the material. Consequently, the SHG (or THG) process can be resonantly enhanced. This phenomenon aligns with observations made in other hybrid metal halide systems,^43,45^ further substantiating our findings.

To confirm the presence of the STE state for the **1·4H**_**2**_**O**, we analyzed the temperature-dependent PL spectra (Figure S13b). The compound exhibits a noticeable PL signal at RT with a broad emission spanning the 450–700 nm range. As the temperature increases, the emission undergoes a red-shift with a decrease in intensity. Additionally, this crystal displays a long lifetime at RT in Figure S14, which is characteristic of self-trapped excitons.^46^ These observations support the existence of the STE state and its relation to structural deformation in the crystal lattice. Overall, our findings confirm the remarkable exciton resonant enhancement behavior of SHG and THG in the supramolecular metal halide system, enabling a wide frequency conversion range that covers a significant portion of the visible spectrum.

#### Structural and Optical Reversibility by Water Vapor Absorption

**Figure 4:**
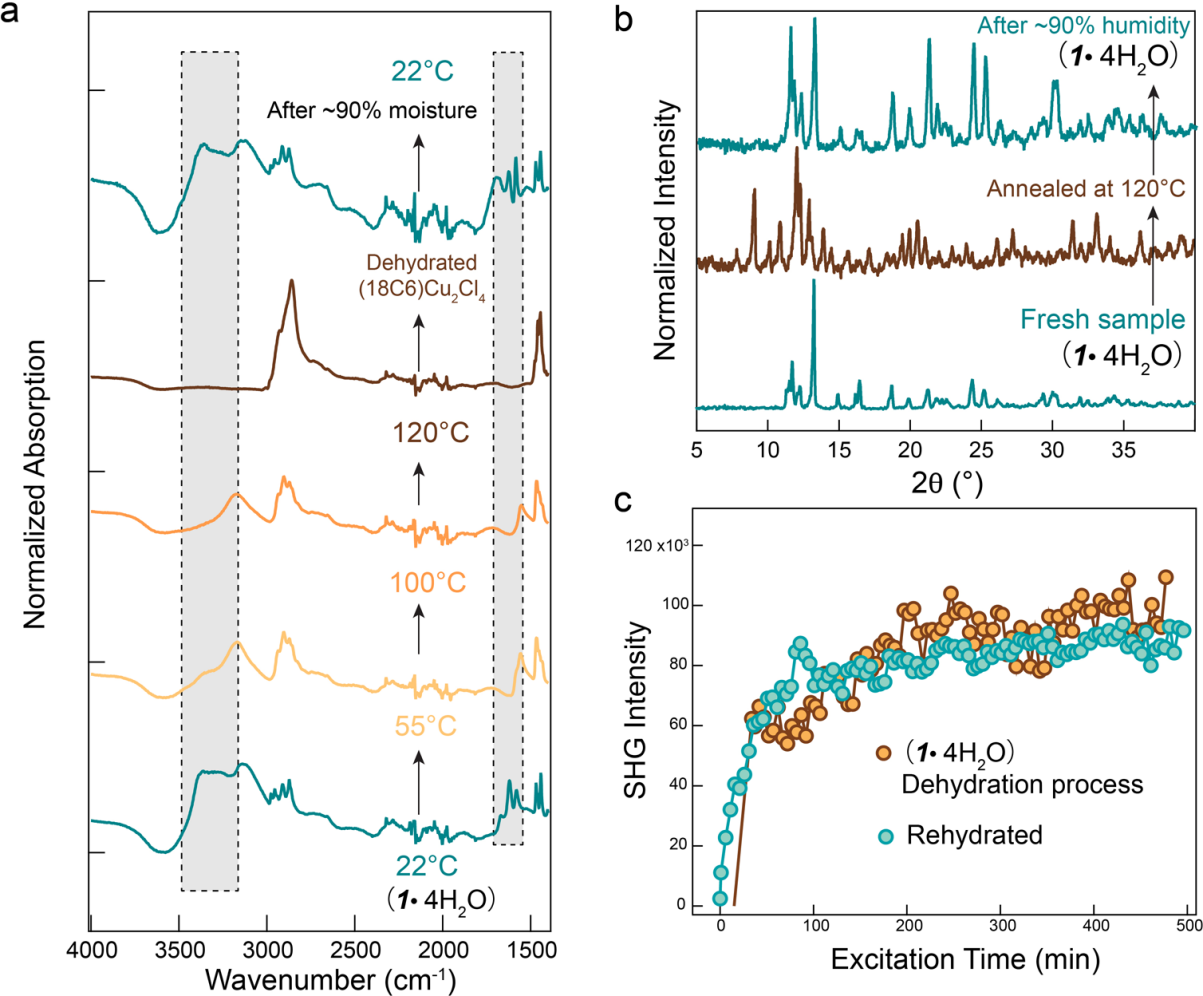
Reversibility of structural and optical properties. (a) In situ FTIR spectrum for **1***·***4H**_**2**_**O** at different temperatures and the after-rehydration spectrum of annealed **1·4H**_**2**_**O**. (b) Reversible PXRD pattern of 120 °C-annealed **1***·***4H**_**2**_**O** under high moisture conditions. (c) Reversible SHG switching properties of rehydrated **1***·***4H**_**2**_**O** after continuous NIR laser illumination.

After examining the thermally annealed form of the **1·4H**_**2**_**O** crystal, we observed that the crystal underwent a color change from brown to blue when exposed to ambient conditions for 2 days, particularly on humid days. This suggests that the dehydration process can be reversed when the sample comes into contact with moisture. In situ Fourier transform infrared spectroscopy (FTIR) and PXRD were used to study the reversibility of the dehydration/rehydration process. The FTIR spectrum (Figure 4a) reveals characteristic absorption peaks in the ranges of 3600–3000 and 1670–1600 cm^–1^ corresponding to the stretching and bending vibration modes of water molecules. Increasing the temperature to 55 and 100 °C resulted in the gradual disappearances of the peaks at approximately 3375 and 1670 cm^–1^. At 120 °C, no signature peaks from water molecules were observed, indicating the progressive loss of water molecules due to thermal effects, consistent with the earlier TGA-DSC and PXRD results. To verify the reversibility of water adsorption and release, we stored the dehydrated sample overnight in a high-moisture environment (∼90% humidity). Along with the color change from brown to blue, the distinct absorption peaks of water molecules reappeared in the FTIR spectrum, providing evidence for the reversible nature of the water absorption process. Moreover, we examined the structural characteristics of the fresh sample, annealed sample, and sample exposed to high moisture conditions by PXRD (Figure 4b). The resultant PXRD pattern after rehydration is consistent with **1***·***4H**_**2**_**O**.

Motivated by this reversibility, we investigated the reversible SHG intensity before and after dehydration, as shown in Figure 4c. The pristine sample was illuminated by a 1060 nm laser with a power of 764 μJ/cm^2^ for approximately 7 h. Subsequently, the laser was turned off, and the sample was stored in a high-moisture environment for 3 days. This process led to the rehydration and deactivation of the sample, restoring its original time-dependent nonlinearity, particularly the pronounced high-intensity SHG. These characteristics provide a novel approach to designing supramolecular metal halide materials with unique NLO switching properties.

## Conclusions

In conclusion, our research is the first to harness supramolecular metal halides, unveiling an unprecedented SHG off–on contrast achieved through water bridge molecule. This highlights the potential for high NLO properties, even at temperatures up to 120 °C, driven by structural transformations. Our structural and thermal analysis reveal that the SHG was activated in three distinct phases: initial lattice expansion at temperatures below 55 °C with minimal SHG progression, a metastable partial dehydration state (55–118 °C) with a modest SHG increase, and a subsequent structural transformation at ∼118 °C, resulting in pronounced SHG augmentation, as evidenced by temperature-dependent SHG measurements. Interestingly, the dehydrated material can reversibly reintegrate water molecules to regenerate the original structure. This capability facilitates a reversible NLO switching mechanism via dehydration and rehydration. While we present a novel crystalline system exhibiting distinctive NLO switching properties, we acknowledge the need for additional follow-up work. This includes theoretical investigations into the dynamic process of dehydration and rehydration of the bridging water molecule. Overall, these findings significantly advance our understanding of the dynamic behavior of materials, paving the way for the rational design of switchable solid-state NLO systems.

## Methods

### Materials

Chemical reagents and solvents were reagent grade and used without further purification. 18-crown-6 ether (TCI), CuCl_**2**_**·**2H_2_O (Sigma-Aldrich), CuCl_**2**_ (Sigma-Aldrich), and methanol (anhydrous, 99.8%).

### Synthesis of Single Crystals

Both single crystals were grown by a slow-evaporation method. **18-crown-6-ether@Cu**_**2**_**Cl**_**4**_**·4H**_**2**_**O** (**1·4H**_**2**_**O**): 3 mmol of CuCl_2_ and 1 mmol of 18-crown-6-ether were dissolved with 3 mL of methanol under RT and left the solution to evaporate in the fume hood overnight, and blue crystals were obtained. **18-crown-6-ether@Cu**_**2**_**Cl**_**4**_**·H**_**2**_**O (2·H**_**2**_**O)**: 2 mmol of CuCl_2_ and 1 mmol of 18-crown-6-ether were dissolved with 3 mL of methanol under RT and placed the vial opened in the glovebox with low moisture and O_2_ overnight, to obtain brown crystals.

### Single-Crystal X-ray Diffraction (SCXRD)

Crystals were centered on the goniometer of a Rigaku Oxford Diffraction Synergy-S diffractometer equipped with a HyPix6000HE detector and operating with Mo *K*_α_ radiation. The data collection routine, unit cell refinement, and data processing were carried out with the program CrysAlisPro.^47^ Both **1·4H**_**2**_**O** and **2·H**_**2**_**O** crystallize in the monoclinic space group *P*2_1_/*n*. The structures were solved using SHELXT^48^ and refined using SHELXL^49^ via Olex2.^50^ Olex2 was used for molecular graphics generation. CIFs of **1·4H**_**2**_**O** (at 100, 223, 298, and 328 K) and **2·H**_**2**_**O** (prepared directly and by heating of **1·4H**_**2**_**O**) have been deposited as CCDC 2298794–2298799 at the Cambridge Crystallographic Data Centre (www.ccdc.cam.ac.uk/data_request/cif).

### Powder X-ray Diffraction (PXRD)

RT PXRD patterns were collected on a Rigaku Miniflex 600 Benchtop Powder XRD instrument with Cu *K*_α_ radiation and a scintillation counter. The *in situ* heating PXRD patterns were measured on a Rigaku Miniflex 6G Benchtop Powder XRD with a Cu *K*_α_ radiation and a HyPix-400MF Hybrid Pixel Array 0D/1D/2D Detector. The temperature was controlled with an Anton-Paar BTS 500 benchtop heating stage. Samples were ground into powder and filled in a 0.2 mm depth stage for measurements. The sample was heated in air.

### Thermal Gravity (TG)–Differential Scanning Calorimetry (DSC) Measurement

The thermal measurements were performed on compounds **1·4H**_**2**_**O** and **2·H**_**2**_**O** using a TA Instruments-TGA 5500 and DSC Q2000, respectively. They were collected at a heating (or cooling) rate of 10 °C/min under nitrogen purge.

### UV–Vis Diffuse Reflectance Measurement

Absorption measurements were performed with an Agilent Technologies Cary 500 UV–vis–NIR spectrophotometer equipped with a diffuse reflectance accessory.

### Fourier Transform Infrared (FTIR) Spectroscopy

FTIR was performed using a Varian 670-IR spectrometer with a DTGS detector using the Pike Technologies GladiATR attachment (diamond crystal). The spectra of the **1·4H**_**2**_**O** powders were collected as an average of 32 scans at a 4 cm^–1^ resolution.

### SHG and PL Measurements

The laser at a frequency of 1 kHz is generated from the Astrella-F-1K one-box femtosecond amplifier with an optical parametric amplifier system. The scattered light from the samples was gathered using a pair of lenses and focused into the fiber, which was then transmitted to the spectrometer with a CCD camera system from Princeton Instruments. A variable ND filter was used to tune the laser power intensity. For the polarized SHG measurement, an extra linear polarizer and an NIR half-wave plate were used before the sample. The PL spectrum excited by a 375 nm laser was recorded using a nitrogen-cooled charge-coupled device camera equipped with a monochromator.

### Dielectric and Polarization-Electric Field Hysteresis Loop Measurements

The P-E hysteresis loops were measured by using the Radiant LC ferroelectric tester with a high-voltage interface and a Trek 609B high-voltage amplifier. The temperature-dependent dielectric constant at 10 kHz was measured using an Agilent 4294A impedance analyzer and a box furnace. The samples for temperature-dependent PE hysteresis loops and dielectric measurements were equipped with silver epoxy electrodes.

### Solid-State PL Lifetime Measurement

Time-resolved photoluminescence measurements were conducted with an Edinburgh Instruments LP980 laser flash photolysis system. The excitation source was a frequency-tripled (355 nm) spectroscopic quantum-ray INDI Nd:YAG laser, operating at 1 Hz with a 6–8 ns pulse width. The spectrometer was equipped with an Andor i-Star ICCD camera for steady-state measurements and a Hamamatsu R928 PMT for measuring single-wavelength kinetics. The reported single-wavelength kinetic lifetimes were averaged over multiple trials, and a long-pass filter with a 400 nm cutoff was used to block ca. 99% of the 355 nm excitation pulses from entering the detection system.

## Supporting Information Available

(Figures S1–S14 and Tables S1–S6) X-ray diffraction measurements; thermal analysis measurements; time-dependent SHG measurements; polarization resolved SHG measurements; electrical P-E hysteresis loop measurements; laser-induced damage threshold measurement; diffused reflectance and PL measurements and time-dependent PL measurements (PDF)
