# Supplementary material for: Supramolecular Metal Halide Complexes for High-Temperature Nonlinear Optical Switches
Source: J Am Chem Soc. 2024 Feb 23;146(13):8971–80. doi: 10.1021/jacs.3c13079 (PMC10996001; doi:10.1021/jacs.3c13079)
Supplement: Supplementary file 1 — ja3c13079_si_001.pdf [file ja3c13079_si_001.pdf]

Supplementary Information

Supramolecular Metal Halide Complexes for High-Temperature Non-linear Optical Switches

Qian Wang[1], Jianbo Jin[3], Zhongxuan Wang[4], Shenqiang Ren[4], Qingyu Ye[1], Yixuan Dou[1], Sunhao Liu[1], Amanda Morris[1], Carla Slebodnick[1], Lina Quan*[1,2]

[1]Department of Chemistry, Virginia Tech, Blacksburg, Blacksburg, Virginia 24060, United States

[2]Department of Materials and Science Engineering, Virginia Tech, Blacksburg, Blacksburg, Virginia 24060, United States

[3]Department of Chemistry, University of California, Berkeley, California 94720, United States

[4]Department of Materials Science and Engineering, University of Maryland, College Park, MD 20742, United States

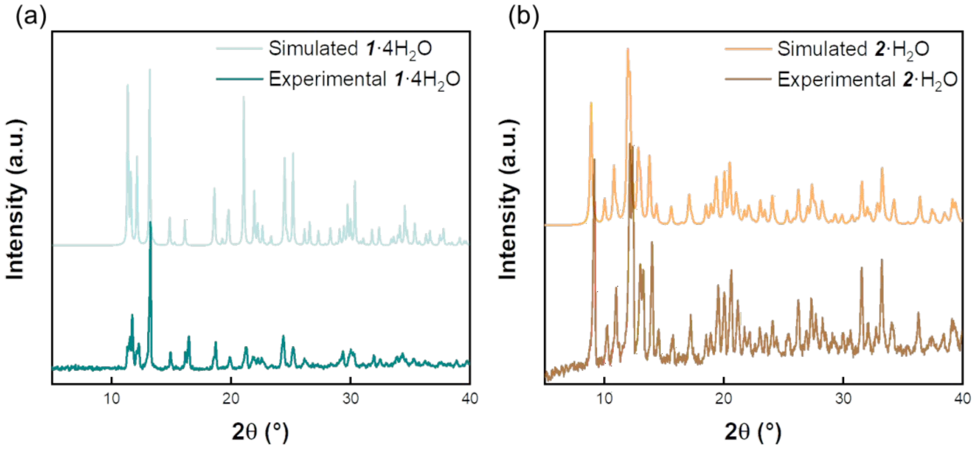

Figure S1. Experimental and calculated PXRD patterns from SCXRD measurement of (a) ***1*·4H₂O** and (b) ***2*·H₂O**.

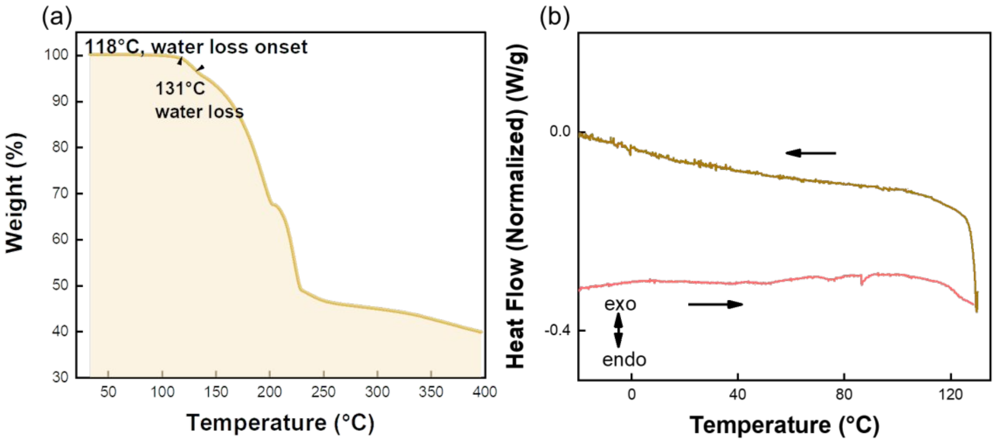

Figure S2. (a) The TGA and (b) DSC curve of ***2*·H_2O**

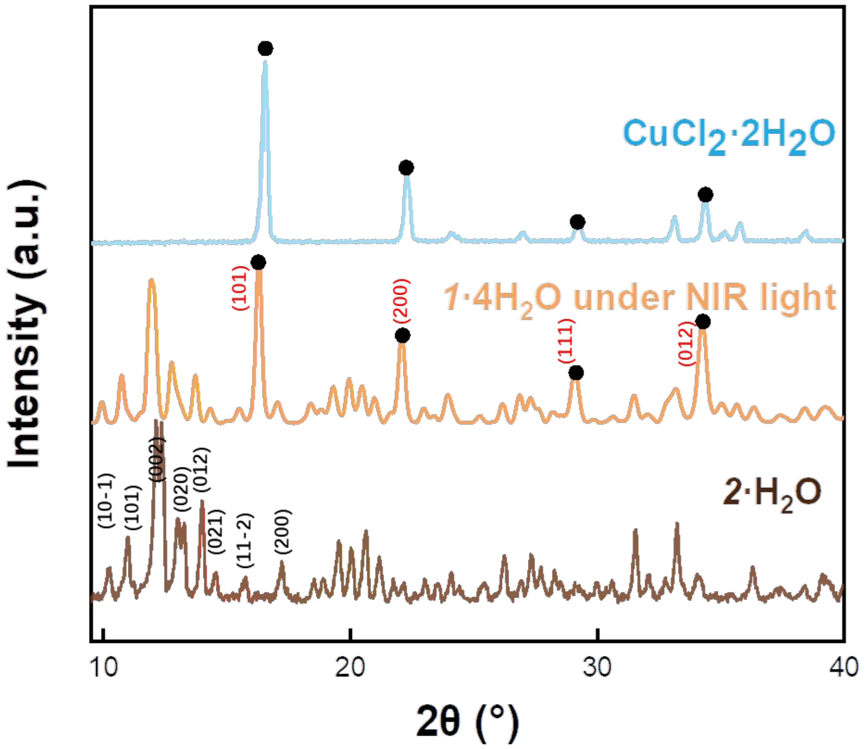

Figure S3. PXRD patterns of $CuCl_2{\cdot}2H_2O$, ***1***$\boldsymbol{\cdot}$**$4H_2O$** under NIR illumination and ***2***$\boldsymbol{\cdot}$**H_2O**. Compared to the pattern of ***2***$\boldsymbol{\cdot}$**H_2O,** four obvious peaks of ***1***$\boldsymbol{\cdot}$**$4H_2O$** zapped by NIR laser illumination are coming from $CuCl_2{\cdot}2H_2O$.

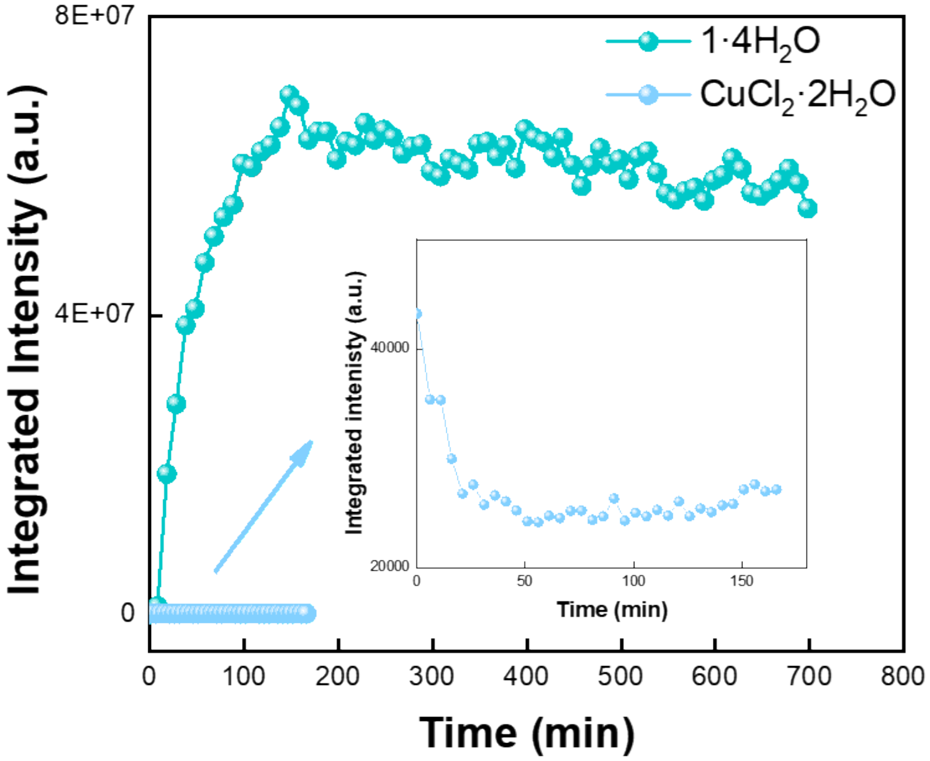

Figure S4. The time-dependent NLO property of ***1*·4H₂O** and $CuCl_2·2H_2O$ under 1060nm excitation at the pump power of 764 μJ/cm^2. SHG signal of ***1*·4H₂O** switches from zero to a huge plateau value with an exceptional off-on contrast after around 100 minutes NIR excitation while $CuCl_2·2H_2O$ (the inset zoom-in image) displays a negligible SHG signal. [1,2]

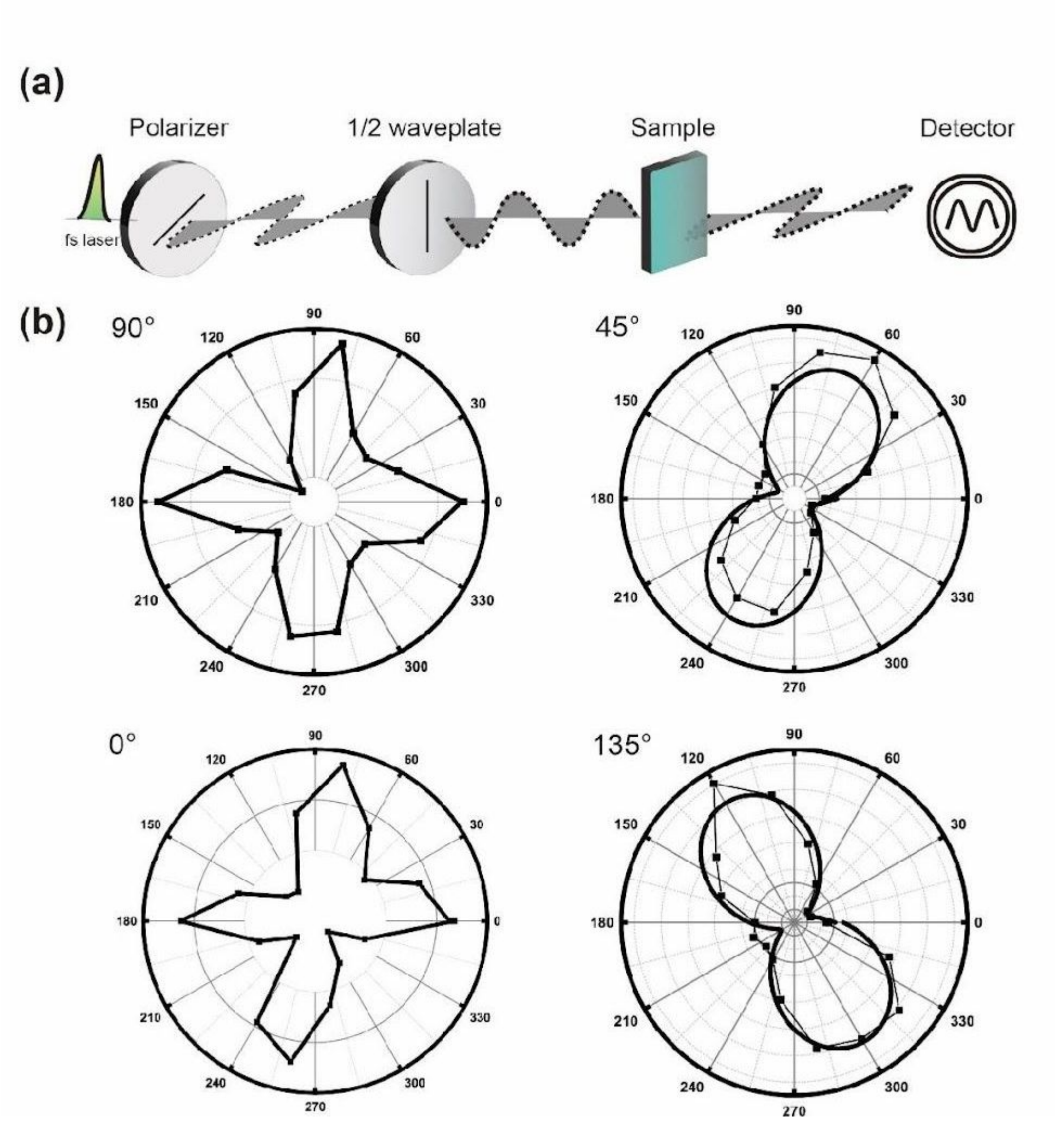

Figure S5. (a) The setup for rotational anisotropy SHG measurement. (b) Rotational anisotropy SHG measurement at different azimuth. It can be clearly seen that these patterns evolve with the azimuth changing, where azimuth is the rotation angle of sample relative to its initial position. This measurement was done by rotating the1/2 λ waveplate in front of the sample. At each azimuth angle, the saturated ***1*·4H₂O** has a fully polarized angle dependent SHG.

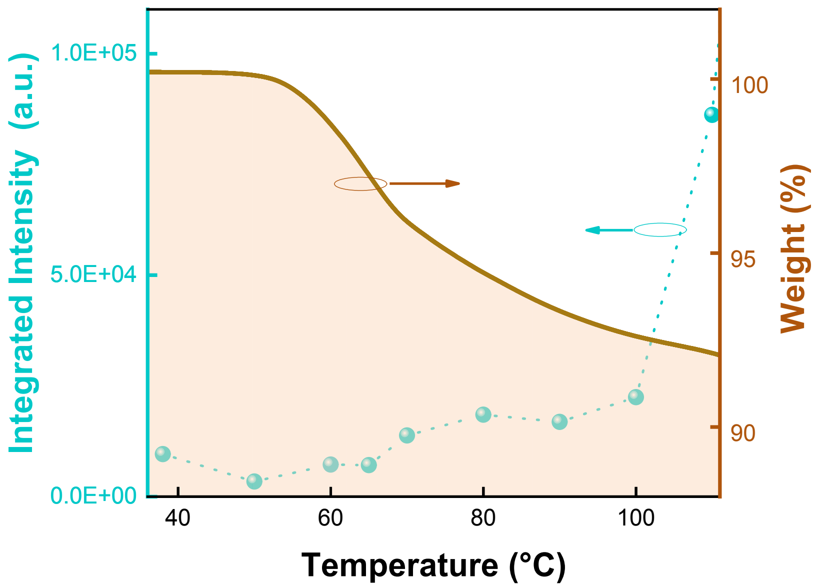

Figure S6. Temperature-dependent SHG of ***1***·**4H$_2$O** in the temperature range of 60-110°C (blue) and TGA curve (brown). It shows gradual SHG growth as a function of temperature.

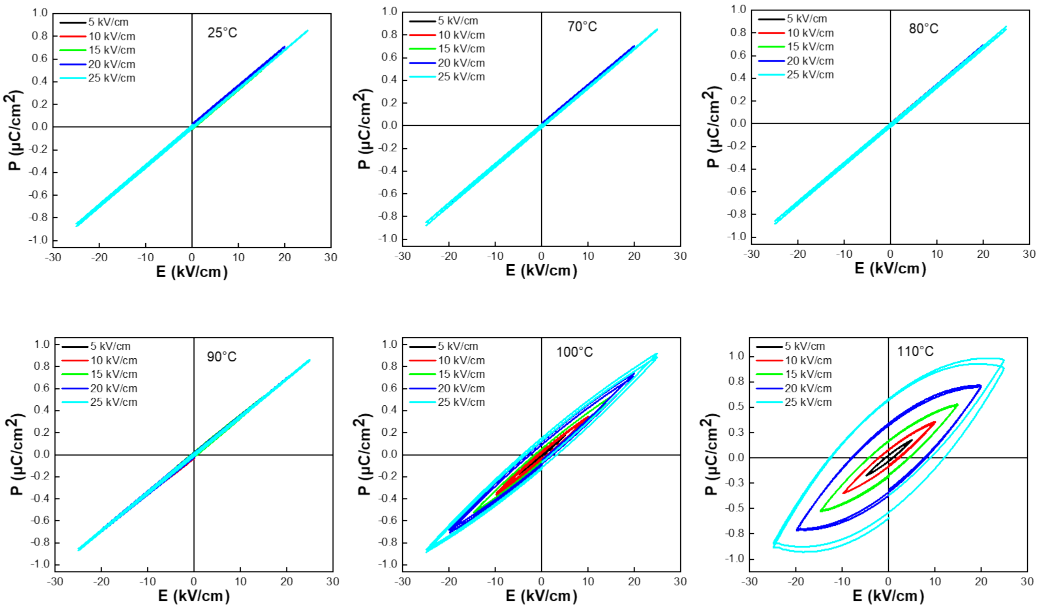

Figure S7. The P-E hysteresis loop shows the polarity in ***1*·4H₂O** at different temperature from 25°C to 110°C. Polarization emerges at 100°C.

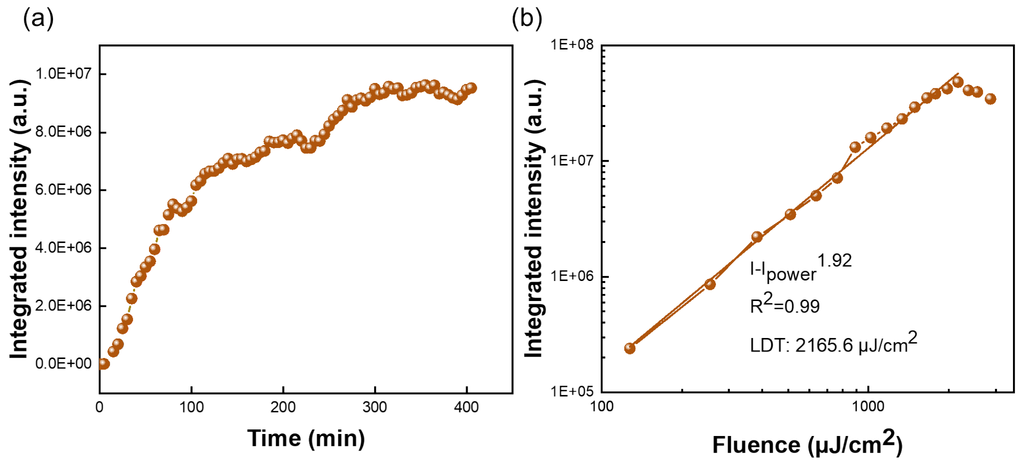

Figure S8. Nonlinear SHG properties of ***2*·H_2O.** (a) Time-dependent SHG of ***2*·H_2O** under 1060nm femtosecond laser of 764 μJ/cm^2. (b) Quadratic relationship of ***2*·H_2O** with a laser-induced damage threshold (LIDT) of 2165.6 μJ/cm^2.

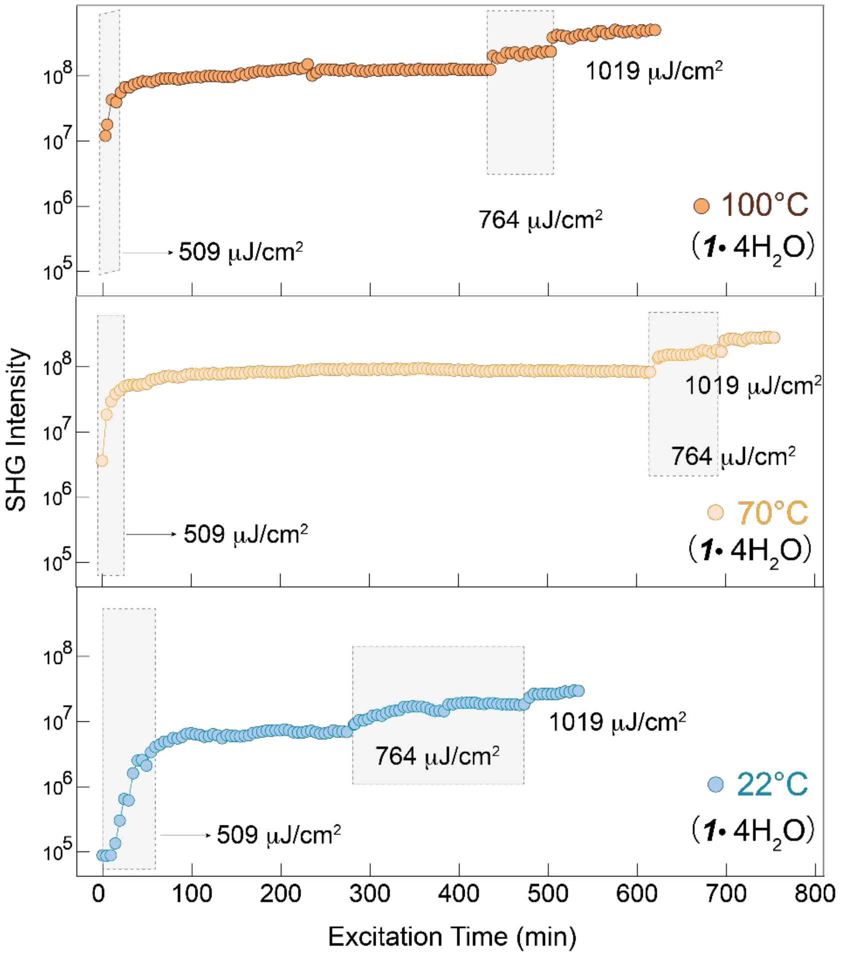

Figure S9. In-situ time-dependent SHG enhancement of ***1*·4H₂O** and corresponding effects by altering the excitation power density and temperature. At each temperature, the sample was illuminated by 509 μJ/cm², 704 μJ/cm² and 1019 μJ/cm² in sequence for a certain time until the SHG intensity level off.

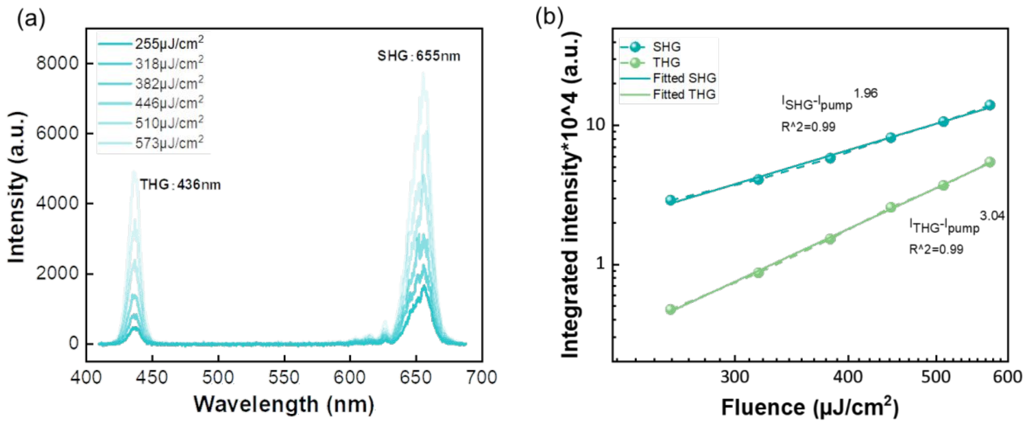

Figure S10. (a) Power dependent spectrum of ***1*·4H$_2$O** excited by 1310nm femtosecond laser. (b) The fitted quadratic and cubic power law for ***1*·4H$_2$O.**

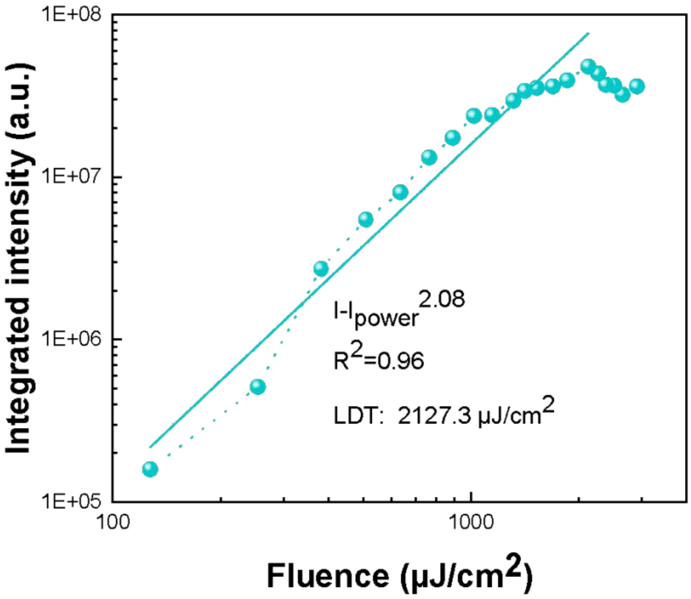

Figure S11. Quadratic nonlinear relationship of ***1*·4H₂O** with a laser-induced damage threshold (LIDT) of 2127.3 μJ/cm^2.

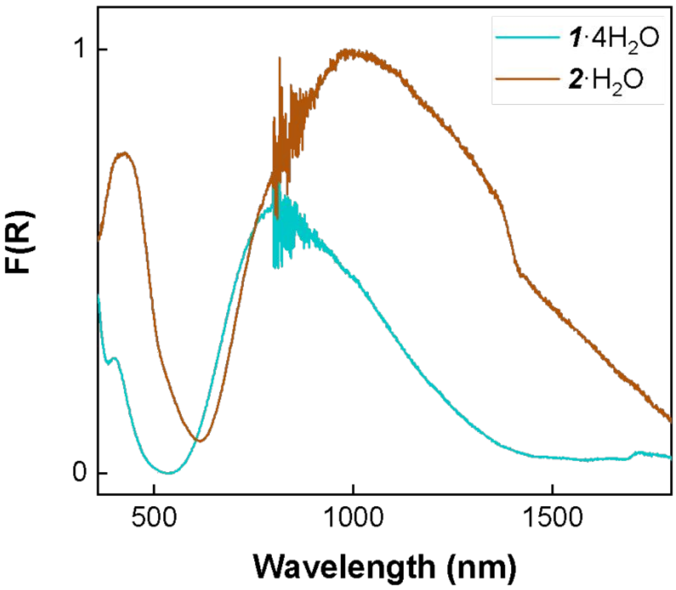

Figure S12. Diffused reflectance spectra of ***1***·**4H$_2$O** and ***2***·**H$_2$O**, the reflectance in NIR region is correspondence to the Cu^{2+} d-d transition.

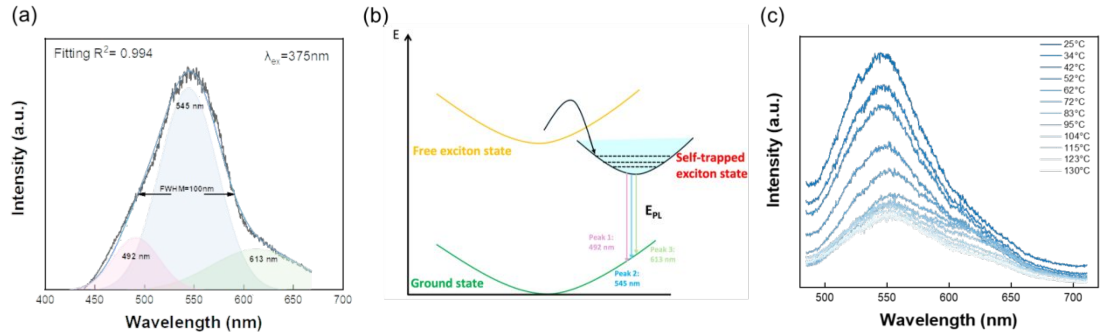

Figure S13. (a) Room temperature PL spectrum of ***1*·4H₂O** under 375nm excitation. (b) Schematic of the energy level structure of STE. (c) Temperature dependent PL of ***1*·4H₂O** excited by 375nm laser.

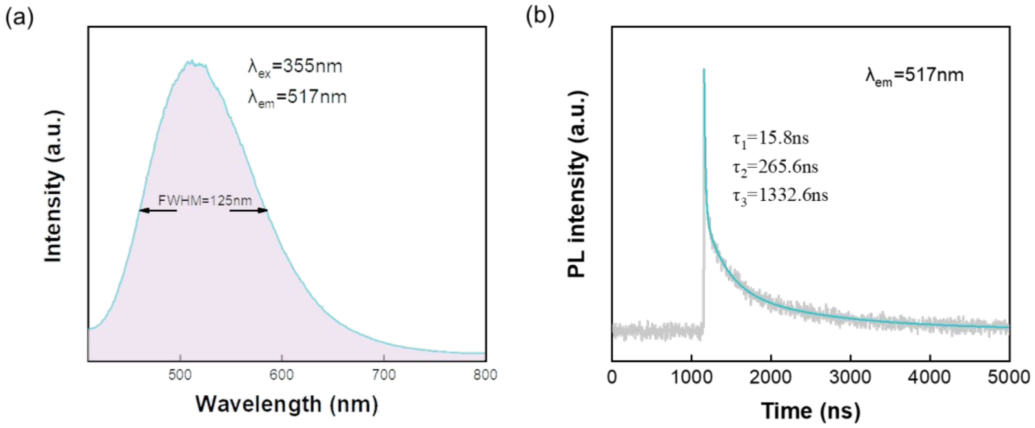

Figure S14. (a) Room temperature PL spectrum of ***1*·4H₂O** excited by 355nm continuous wave laser. (b) Room temperature transient PL decay curve of ***1*·4H₂O** monitored at 517nm and excited by 355nm laser. τ_1 is coming from the instrument response time.

Table S1: Crystal data and structure refinement for *1*·4H_2O measured at 100K.

Empirical formula	$C_{12}H_{32}Cl_4Cu_2O_{10}$
Formula weight	605.25
Temperature/K	99.98(12)
Crystal system	monoclinic
Space group	$P2_1/n$
a/Å	8.95290(10)
b/Å	9.53380(10)
c/Å	13.4104(2)
α/°	90
β/°	92.6610(10)
γ/°	90
Volume/Å^3	1143.41(2)
Z	2
ρ_{calc}g/cm^3	1.758
μ/mm^{-1}	2.371
F(000)	620.0
Crystal size/mm^3	0.199 × 0.11 × 0.04
Radiation	Mo Kα (λ = 0.71073)
2Θ range for data collection/°	5.244 to 76.69
Index ranges	$-15 \le h \le 14$, $-16 \le k \le 16$, $-22 \le l \le 23$
Reflections collected	45507
Independent reflections	6020 [R_{int} = 0.0346, R_{sigma} = 0.0206]
Data/restraints/parameters	6020/0/147
Goodness-of-fit on F^2	1.046
Final R indexes [I>=2σ (I)]	R_1 = 0.0198, wR_2 = 0.0450
Final R indexes [all data]	R_1 = 0.0240, wR_2 = 0.0461
Largest diff. peak/hole / e Å^{-3}	0.52/-0.35

Table S2: Crystal data and structure refinement for *1*·4H_2O measured at 223K.

Empirical formula	$C_{12}H_{32}Cl_4Cu_2O_{10}$
Formula weight	605.25
Temperature/K	222.99(16)
Crystal system	monoclinic
Space group	$P2_1/n$
a/Å	8.9846(2)
b/Å	9.5595(2)
c/Å	13.5452(4)
α/°	90
β/°	92.688(2)
γ/°	90

Volume/Å^3	1162.10(5)
Z	2
ρ_{calc}g/cm^3	1.730
μ/mm^{-1}	2.333
F(000)	620.0
Crystal size/mm^3	0.181 × 0.072 × 0.062
Radiation	Mo Kα (λ = 0.71073)
2Θ range for data collection/°	5.218 to 72.634
Index ranges	$-14 \le h \le 14, -15 \le k \le 15, -22 \le l \le 22$
Reflections collected	23811
Independent reflections	5617 [R_{int} = 0.0311, R_{sigma} = 0.0280]
Data/restraints/parameters	5617/0/147
Goodness-of-fit on F^2	1.039
Final R indexes [I>=2σ (I)]	R_1 = 0.0276, wR_2 = 0.0588
Final R indexes [all data]	R_1 = 0.0401, wR_2 = 0.0618
Largest diff. peak/hole / e Å^{-3}	0.67/-0.40

Table S3: Crystal data and structure refinement for *1*·4H_2O measured at 298K.

Empirical formula	$C_{12}H_{32}Cl_4Cu_2O_{10}$
Formula weight	605.25
Temperature/K	298.0(3)
Crystal system	monoclinic
Space group	P2$_1$/n
a/Å	9.0019(3)
b/Å	9.5736(3)
c/Å	13.6552(5)
α/°	90
β/°	92.561(3)
γ/°	90
Volume/Å^3	1175.64(7)
Z	2
ρ_{calc}g/cm^3	1.710
μ/mm^{-1}	2.306
F(000)	620.0
Crystal size/mm^3	0.181 × 0.072 × 0.062
Radiation	Mo Kα (λ = 0.71073)
2Θ range for data collection/°	5.198 to 72.632
Index ranges	$-15 \le h \le 15, -15 \le k \le 15, -22 \le l \le 22$
Reflections collected	24218
Independent reflections	5679 [R_{int} = 0.0355, R_{sigma} = 0.0331]

Data/restraints/parameters	5679/0/147
Goodness-of-fit on F^2	1.028
Final R indexes [I>=2σ (I)]	R_1 = 0.0341, wR_2 = 0.0717
Final R indexes [all data]	R_1 = 0.0572, wR_2 = 0.0775
Largest diff. peak/hole / e $Å^{-3}$	0.82/-0.52

Table S4. Crystal data and structure refinement for *1*·4H_2O measured at 328K.

Empirical formula	$C_{12}H_{32}Cl_4Cu_2O_{10}$
Formula weight	605.25
Temperature/K	328.0(3)
Crystal system	monoclinic
Space group	$P2_1/n$
a/Å	9.0099(4)
b/Å	9.5715(5)
c/Å	13.7164(7)
α/°	90
β/°	92.477(4)
γ/°	90
Volume/$Å^3$	1181.77(10)
Z	2
$\rho_{calc}g/cm^3$	1.701
μ/mm^{-1}	2.294
F(000)	620.0
Crystal size/mm^3	0.181 × 0.072 × 0.062
Radiation	Mo Kα (λ = 0.71073)
2Θ range for data collection/°	6.214 to 61.012
Index ranges	-12 ≤ h ≤ 12, -13 ≤ k ≤ 13, -18 ≤ l ≤ 19
Reflections collected	15026
Independent reflections	3597 [R_{int} = 0.0428, R_{sigma} = 0.0425]
Data/restraints/parameters	3597/0/138
Goodness-of-fit on F^2	1.030
Final R indexes [I>=2σ (I)]	R_1 = 0.0365, wR_2 = 0.0726
Final R indexes [all data]	R_1 = 0.0604, wR_2 = 0.0790
Largest diff. peak/hole / e $Å^{-3}$	0.61/-0.48

Table S5. Crystal data and structure refinement for *1*·4H_2O annealed at 120 °C

Empirical formula	$C_{24}H_{52}Cl_8Cu_4O_{14}$
Formula weight	1102.41
Temperature/K	298.0(3)

Crystal system	monoclinic
Space group	$P2_1/n$
a/Å	10.4492(2)
b/Å	13.7218(3)
c/Å	14.7121(3)
α/°	90
β/°	95.830(2)
γ/°	90
Volume/Å^3	2098.54(9)
Z	2
ρ_{calc}g/cm^3	1.745
μ/mm^{-1}	2.564
F(000)	1120.0
Crystal size/mm^3	0.2 × 0.17 × 0.01
Radiation	Mo Kα (λ = 0.71073)
2Θ range for data collection/°	7.116 to 76.516
Index ranges	$-17 \leq h \leq 17, -23 \leq k \leq 23, -24 \leq l \leq 24$
Reflections collected	54297
Independent reflections	11039 [R_{int} = 0.0383, R_{sigma} = 0.0333]
Data/restraints/parameters	11039/78/282
Goodness-of-fit on F^2	1.021
Final R indexes [I>=2σ (I)]	R_1 = 0.0332, wR_2 = 0.0687
Final R indexes [all data]	R_1 = 0.0637, wR_2 = 0.0761
Largest diff. peak/hole / e Å^{-3}	0.40/-0.33

Table S6. Crystal data and structure refinement for *2*·H_2O at 100K.

Empirical formula	$C_{24}H_{52}Cl_8Cu_4O_{14}$
Formula weight	1102.41
Temperature/K	100.15
Crystal system	monoclinic
Space group	$P2_1/n$
a/Å	10.2884(3)
b/Å	13.5966(3)
c/Å	14.6451(4)
α/°	90
β/°	95.913(3)
γ/°	90
Volume/Å^3	2037.76(9)
Z	2
ρ_{calc}g/cm^3	1.797
μ/mm^{-1}	2.641

F(000)	1120.0
Crystal size/mm^3	0.13 × 0.09 × 0.04
Radiation	Mo Kα (λ = 0.71073)
2Θ range for data collection/°	6.614 to 76.482
Index ranges	−17 ≤ h ≤ 17, −23 ≤ k ≤ 22, −24 ≤ l ≤ 24
Reflections collected	39529
Independent reflections	10651 [R_{int} = 0.0592, R_{sigma} = 0.0634]
Data/restraints/parameters	10651/30/253
Goodness-of-fit on F^2	1.025
Final R indexes [I>=2σ (I)]	R_1 = 0.0395, wR_2 = 0.0696
Final R indexes [all data]	R_1 = 0.0725, wR_2 = 0.0764
Largest diff. peak/hole / e $Å^{-3}$	0.78/-0.77

References

1 Boeré, R. T. Crystal Structures of $CuCl_2 \cdot 2H_2O$ (Eriochalcite) and $NiCl_2 \cdot 6H_2O$ (Nickelbischofite) at Low Temperature: Full Refinement of Hydrogen Atoms Using Non-Spherical Atomic Scattering Factors. *Crystals* 2023, **13**, 293.

2 Halcrow, M. A. Jahn-Teller distortions in transition metal compounds, and their importance in functional molecular and inorganic materials. *Chem Soc Rev* 2013, **42**, 1784-1795.
